# Mesenchymal-specific Alms1 knockout in mice recapitulates metabolic features of Alström syndrome

**DOI:** 10.1016/j.molmet.2024.101933

**Published:** 2024-04-06

**Authors:** Eleanor J. McKay, Ineke Luijten, Xiong Weng, Pablo B. Martinez de Morentin, Elvira De Frutos González, Zhanguo Gao, Mikhail G. Kolonin, Lora K. Heisler, Robert K. Semple

**Affiliations:** 1 Centre for Cardiovascular Science, University of Edinburgh, Edinburgh, UK; 2 The Rowett Institute, University of Aberdeen, Aberdeen, UK; 3 School of Biomedical Sciences, Faculty of Biological Sciences, University of Leeds, Leeds, UK; 4 Área de Fisiología Humana, Departamento de Ciencias básicas de la Salud, Facultad de ciencias de la Salud, Universidad Rey Juan Carlos, 28922 Alcorcón, Madrid, Spain; 5 Institute of Molecular Medicine, University of Texas Health Sciences Center at Houston, Houston, TX 77030, USA; 6 MRC Human Genetics Unit, Institute of Genetics and Cancer, University of Edinburgh, Edinburgh, UK

**Keywords:** Alms1, Alström syndrome, Insulin resistance, Diabetes, Adipose tissue, Mouse

## Abstract

**Objective:**

Alström Syndrome (AS), caused by biallelic *ALMS1* mutations, includes obesity with disproportionately severe insulin resistant diabetes, dyslipidemia, and fatty liver. Prior studies suggest that hyperphagia is accounted for by loss of ALMS1 function in hypothalamic neurones, whereas disproportionate metabolic complications may be due to impaired adipose tissue expandability. We tested this by comparing the metabolic effects of global and mesenchymal stem cell (MSC)-specific Alms1 knockout.

**Methods:**

Global *Alms1* knockout (KO) mice were generated by crossing floxed *Alms1* and CAG-Cre mice. A *Pdgfrα*-Cre driver was used to abrogate Alms1 function selectively in MSCs and their descendants, including preadipocytes. We combined metabolic phenotyping of global and *Pdgfrα*+ *Alms1*-KO mice on a 45% fat diet with measurements of body composition and food intake, and histological analysis of metabolic tissues.

**Results:**

Assessed on 45% fat diet to promote adipose expansion, global *Alms1* KO caused hyperphagia, obesity, insulin resistance, dyslipidaemia, and fatty liver. *Pdgfrα*-*cre* driven KO of *Alms1* (MSC KO) recapitulated insulin resistance, fatty liver, and dyslipidaemia in both sexes. Other phenotypes were sexually dimorphic: increased fat mass was only present in female *Alms1* MSC KO mice. Hyperphagia was not evident in male *Alms1* MSC KO mice, but was found in MSC KO females, despite no neuronal Pdgfr*α* expression.

**Conclusions:**

Mesenchymal deletion of *Alms1* recapitulates metabolic features of AS, including fatty liver. This confirms a key role for *Alms1* in the adipose lineage, where its loss is sufficient to cause systemic metabolic effects and damage to remote organs. Hyperphagia in females may depend on Alms1 deficiency in oligodendrocyte precursor cells rather than neurones. AS should be regarded as a *forme fruste* of lipodystrophy.

## Highlights

• We tested whether lipotoxic insulin resistance in Alström Syndrome (AS) is due to adipose “failure”.
• *Alms1* KO in mesenchymal stem cells (MSCs) recapitulated insulin resistance, adipose hypertrophy and fatty liver.
• Surprisingly, hyperphagia was seen in females with neurone-sparing, MSC-specific *Alms1* KO.
• Our findings support a key role for constrained adipose expansion in metabolic complications of AS.
• Oligodendrocyte precursor cells may play a role in the hyperphagic obesity of AS.

## 1 Introduction

Alström syndrome (AS), caused by biallelic loss-of-function *ALMS1* mutations, features childhood-onset retinal degeneration, deafness, obesity, diabetes mellitus, and cardiomyopathy [1]. *ALMS1* encodes a 460 kDa protein localised to centrosomes and basal bodies of primary cilia [2,3] but its function remains unknown.

Birthweight in AS is normal, but 98% of affected people develop truncal obesity within one year [4,5]. Insulin resistance (IR) in AS is common, usually of childhood onset [4,6], and reportedly five times more severe than in BMI-matched controls [7]. It is associated with severe hypertriglyceridemia, low serum HDL cholesterol [[8], [9], [10], [11]], fatty liver and its sequelae [[4], [5], [6], [7]], and diabetes. Diabetes, usually of childhood or early adult onset, occurs in 68% of people with AS [4,6,7]. Understanding the pathogenesis of the severe, early onset metabolic complications of AS is crucial to improving care for affected people. As the metabolic profile of AS phenocopies common obesity-related metabolic disease in exaggerated form, unpicking the underlying mechanisms may also yield important insights into common disease.

ALMS1 is globally expressed, but the metabolic profile of AS is reminiscent of the dyslipidemic severe IR and fatty liver of lipodystrophy. This has led to the hypothesis that metabolic complications of AS are a form of “adipose failure” [6]. This could be attributed to impaired adipocyte differentiation, or attenuation of the mitotically active adipocyte precursor pool. This is plausible given implication of primary cilia in early preadipocyte differentiation [12]. A recent murine study, however, suggested that reactivation of *Alms1* in postmitotic, mature adipocytes mitigates the metabolic consequences of global *Alms1* knockout [13]. While adding to evidence that adipose *ALMS1* deficiency may explain crucial metabolic features of AS, this was surprising, as it implicated ALMS1 in function of non ciliated mature adipocytes.

We set out to test whether dysmetabolism in AS is attributable to loss of *Alms1* in the adipose lineage using a complementary strategy. We generated global *Alms1* knockout (KO) and mesenchymal-specific *Alms1* KO lines by crossing the same floxed Alms1 line with *CAG-cre* or *Pdgfrα-cre* mice [[14], [15], [16]] and compared the metabolic consequences. Pdgfrα-cre targets preadipocytes and their descendants, but endogenous *Pdgfrα* is more accurately viewed as mesenchymal stem cell (MSC) marker [17]. Importantly, *Pdgfrα*-Cre driven recombination occurs only at very low levels in liver [16] and skeletal muscle [15,16]. Thus, the two major peripheral metabolic organs apart from adipose tissue are largely untargeted by *Pdgfrα* promoter-driven Cre expression. This enables testing of the role of adipose *Alms1* in metabolic complications of AS.

## 2 Materials and methods

### 2.1 Animal origin and generation

*Alms1*^tm1c(EUCOMM)Hmgu^ (*Alms1*^tm1c^) mice, with floxed exon 7 of *Alms1,* were purchased from GenOway. CAG-Cre mice [18] gifted by Dr Matthew Brook were used to generate global *Alms1* knockout (KO) mice without potentially confounding Cre expression. *Pdgfrα*-Cre mice [14] from The Jackson Laboratory (#013148) were used to generate *Alms1* mesenchymal stem cell (MSC-) KO mice. Wild type (WT) littermate controls for *Alms1* MSC-KO mice expressed *Cre* heterozygously, like their KO littermates. Mice were maintained on a C57/BL6/N background, confirmed by single nucleotide polymorphism profiling (Transnetyx). *Pdgfrα*-Cre × mTom/mGFP mice have been described [19]. All protocols were approved by the University of Edinburgh Biological Science Services in compliance with the UK Home Office Scientific Procedure (Animals) Act 1983.

*Alms1* expression is normal in *Alms1*^tm1c^ mice, but *Cre* expression excises exon 7 and introduces a nonsense mutation in exon 8 (Figure S1A). Given no reliable anti-murine Alms1 antibody, KO was validated by quantitative real-time PCR of genomic DNA (qRT-PCR) (Transnetyx) and cDNA from metabolic tissues. PCR amplification of cDNA across exon 7 indicated complete loss of exon 7 in global *Alms1* KO mice, and partial loss in MSC-KO mice (Figure S1B). qRT-PCR of cDNA targeting an amplicon including the *Alms1* exon 6-7 junction (Supplementary methods) in inguinal white adipose tissue (iWAT) and liver corroborated loss of *Alms1* in global KO mice and partial loss in MSC-KO adipose tissue (Figure S1C and D). Differences in liver in MSC-KO mice were not significant, although a trend to reduction was seen in females (Figure S1E and F).

### 2.2 In vivo metabolic phenotyping

At 6 weeks old, mice were transferred from chow to a 45% fat diet (D12451, Research Diets; caloric value 4.73 kcal/g), with water and diet available *ad libitum*. Body mass was measured weekly from 6 to 24 weeks. Body composition was measured by time-domain nuclear magnetic resonance (tdNMR) using the Bruker Minispec Live Mice Analyzer LF50 [20]. Mice were single housed from 13 weeks of age, with food mass measured at 14 and 18 weeks of age, with difference taken as food intake. Linear regression of food intake and lean mass used mass at 14 weeks old. Metabolic efficiency was calculated by dividing energy associated with body mass increase by food intake for the same period, expressed as a percentage. Change in body mass was defined as change in lean mass (kJ) plus change in fat mass (kJ), with fat and lean mass assigned energy content of 39 kJ/g and 5 kJ/g respectively [21].

Unfasted tail vein blood taken at 1100 at 10 and 19 weeks old was used for glucose and insulin assay. At 20 and 22 weeks of age respectively, an Insulin Tolerance Test (ITT) and oral Glucose Tolerance Test (oGTT) were performed, with lean mass measured one day beforehand. Mice were fasted from 0700 until testing at 1100. For ITT, 1.75 mIU intraperitoneal Humulin S/g lean mass was administered for females, and 2.5 mIU/g for males. For oGTT, 4 mg glucose/g lean mass was delivered by oral gavage as 25% d-glucose in saline. Area of glycaemia curve (AOC) for oGTT and ITT were calculated for each individual animal, with Basal (t0) glycaemia was taken as baseline for area of curve calculations [22] (above and below basal respectively).

### 2.3 Blinding and statistical analysis

Experimenter and analyser were blinded to genotype where possible, including for *in vivo* studies, dissections, tissue processing and analysis. cDNA synthesis and qRT-PCR were unblinded to enable pooling of reverse transcriptase-negative reactions. Unblinding of data was automated in Microsoft Excel to prevent genotype memorisation. Statistical analysis was performed in GraphPad Prism 9.2.0. Normal distribution was assumed for all data, as the small numbers required by best practice in animal research preclude testing for normality. Student's t-test was used to compare data sets pairwise, and ANOVA for comparison of more than 2 groups. Linear regression was used to compare relationships between two dependent variables (e.g. food intake and lean mass) between groups. Bonferroni correction was applied when multiple t-tests were performed on the same graph. Šídák's multiple comparisons test was applied following ANOVA when the animals were studied at multiple time points. Tukey's multiple comparison test was applied following ANOVA to compare values among multiple groups. Insulin values alone were log transformed for analysis.

## 3 Results

### 3.1 Generation of global and mesenchymal stem cell-specific Alms1 knockout mice

To test whether lipotoxic IR in AS is explained by *Alms1* deficiency in the MSC/preadipocyte/adipocyte lineage, mice with global or mesenchymal stem cell (MSC-) specific *Alms1* knockout (KO) were generated. This was achieved using mice expressing *Cre* constitutively (CAG-Cre) for global KO, and in *Pdgfrα*-expressing cells only [[14], [15], [16]] for MSC-KO.

### 3.2 MSC-specific Alms1 deficiency has sexually dimorphic effects on appetite

**Figure 1:**
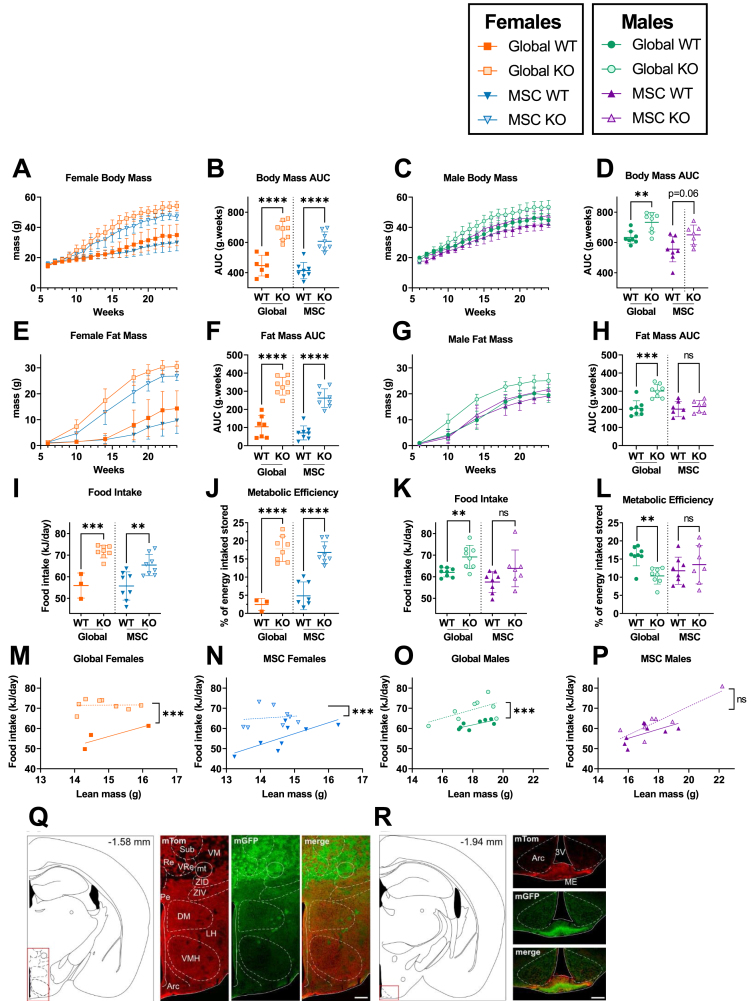
**Female, but not male, MSC-specific *Alms1* knockout mice recapitulate the obesity and hyperphagia of global *Alms1* knockout.** Longitudinal analysis of body mass (A,C) and fat mass (E,G) assessed by td-NMR for female (A,E) and male (C,G) mice on high fat diet. (I–P) Food intake and metabolic efficiency of animals measured from 14 to 18 weeks of age. (M–P) Food intake plotted against lean mass at 14 weeks of age. Comparisons of global WT and KO and MSC WT and KO were performed with identical design at different times, reflected in the dotted line separating comparisons. Longitudinal series (A,C,E,G) plot mean ± sd for each time point. Data points in (B,D,F,H–P) represent individual animals with bars in (B,D,F,H–L) representing mean ± sd, and lines in linear regression graphs (M–P) representing lines of best fit. Comparison between WT and KO in (A–L) was performed using an unpaired two-tailed Student's t-test with Bonferroni correction. Comparison between lines of best fit was performed by simple linear regression, with square brackets showing comparison of y intercepts. No significant change was seen between gradients. ∗∗ denotes p < 0.01, ∗∗∗ denotes p < 0.001 and ∗∗∗∗ denotes p < 0.0001. For females N = 7, 8, 8 and 8 for global WT, global KO, MSC WT and MSC KO respectively, except in food intake studies when many global WT females shred the diet, resulting in N = 3. For males N = 8, 8, 7 and 7 for global WT, global KO, MSC WT and MSC KO respectively. AUC = area under curve. (Q,R) Illustration of coronal plane and representative microphotographs of brain sections from female Pdgfrα-Cre x mTom/mGFP mice showing expression of mTom, mGFP and merged images at bregma levels (Q) −1.58; (R) −1.94. Arc: arcuate hypothalamic nucleus; DM: dorsomedial hypothalamic nucleus; LH: Lateral hypothalamic area; mt: mammillothalamic tract; Pe: periventricular hypothalamic nucleus; Sub: supratrigeminal nucleus; VM: ventromedial thalamic nucleus; VRe: ventral reuniens thalamic nucleus; ZID: zona incerta, dorsal part; ZIV: zona incerta, ventral part; 3 V: Third ventricle. Scale bar 200 μm for Q and 100 μm for R.

To ensure vigorous adipocyte recruitment, 45% HFD was used *ad libitum* from 6 weeks of age. In agreement with published models, male and female global *Alms1* KO mice had higher body mass than WT controls (Figure 1A–D). Female *Alms1* MSC KO mice recapitulated this (Figure 1A,B), but male *Alms1* MSC KO mice showed only a similar trend (Figure 1C,D). Increased body mass of female global- and MSC-KO mice and male global KO mice was accounted for by increased fat mass only (Figures 1E–H, S2A–D).

As expected, both female and male global *Alms1* KO mice were hyperphagic, assessed from 14 to 18 weeks of age (Figure 1I,K). This remained significant when lean body mass was taken into account (Figure 1M,O). Unexpectedly, given neuronal sparing of *Pdgfrα*-Cre, female *Alms1* MSC KO mice, but not males, were also hyperphagic (Figure 1N,P). Increased metabolic efficiency, defined as the percentage of ingested energy stored as lean or fat mass, was seen in female *Alms1* global- and MSC- KO mice (Figure 1J,L). Male global *Alms1* KO mice showed decreased metabolic efficiency, with no difference between male MSC KO and controls (Figure 1J,L).

Given the surprising hyperphagia of female mice with *Alms1* KO limited to *Pdgfrα*-expressing lineages, we next examined brains from *Alms1* WT, *Pdgfrα*-Cre mice harbouring an mGFP/mTomato reporter cassette. This expresses mTomato by default, switching to mGFP on Cre exposure. Evidence of recombination and thus *Pdgfrα-*Cre expression at some point in development was detected in several brain regions without sexual dimorphism. Extremely low recombination rates were seen in regions classically associated with feeding behaviour including the hypothalamic dorsomedial, ventromedial, lateral or arcuate nuclei (Figure 1Q,R). As previously reported [23], a population of recombined cells was visualised in the midline of the median eminence (Figure 1R). Recombination was also seen in the reuniens thalamic nucleus (Figure 1Q), the rostral periolivary region and intermediate nucleus of the lateral lemincus (Figure S1E), anterodorsal thalamic nucleus (Figure S1F), superior cerebellar peduncle (Figure S1G) and area postrema (Figure S1H). Low level co-expression of mTomato and mGFP in some regions could be explained by transient developmental expression of *Pdgfrα*, while scattered cells lacking both mTomato and mGFP are consistent with observations made during generation of the mTom/mGFP reporter model [24]. These findings collectively are in keeping with the expected expression of *Pdgfrα* in oligodendrocyte precursor cells (OPCs) but not neurones.

### 3.3 MSC-specific Alms1 deficiency is sufficient to induce dyslipidemic insulin resistance

**Figure 2:**
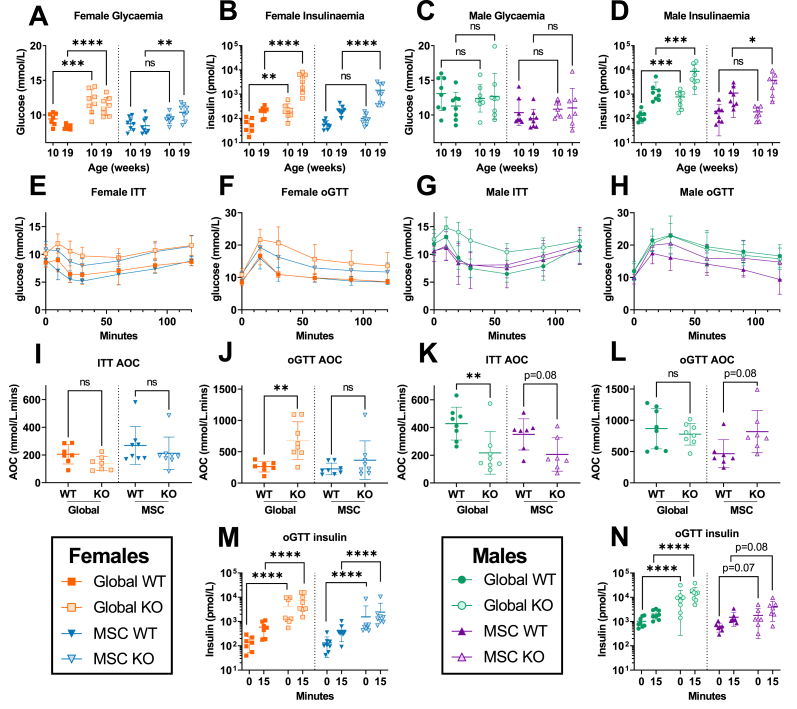
**Mesenchymal stem cell-specific *Alms1* knockout recapitulate the insulin resistance of global *Alms1* loss.** (A–D) Daytime non-fasted blood glucose and insulin concentrations at 10 and 19 weeks of age. (E,G) Insulin tolerance tests (ITT) and (F,H) oral glucose tolerance tests (oGTT). Global WT/KO and MSC WT/KO experiments were performed with identical design at different times, reflected in the dotted line separating comparisons in (A–D, I–N). oGTT and ITT graphs (E–H) show mean ± sd for each time point. All other graphs (A–D, I–N) plot data points representing individual animals, with bars representing mean ± sd. Comparisons of log10 oGTT insulin values (B,D,M,N) and glucose concentration (A,C) were performed by two-way ANOVA with Šídák's multiple comparisons test. Comparison of all other data (I–L) used an unpaired two-tailed Student's t-test with Bonferroni correction. ∗ denotes p < 0.05, ∗∗ denotes p < 0.01, ∗∗∗ denotes p < 0.001 and ∗∗∗∗ denotes p < 0.0001. For females N = 7, 8, 8 and 8 for global WT, global KO, MSC WT and MSC KO respectively. For males N = 8, 8, 7 and 7 for global WT, global KO, MSC WT and MSC KO respectively. AOC = area of the curve; ns = not significant.

To assess for metabolic complications of AS, we first determined non fasting blood glucose and insulin concentrations at 10 and 19 weeks of age. Female global *Alms1* KO mice were hyperglycaemic at both timepoints, and MSC-KO mice only at 19 weeks (Figure 2A), with plasma insulin concentrations markedly increased whenever hyperglycemia was observed (Figure 2B). Male mice showed no hyperglycemia at 10 or 19 weeks (Figure 2C), but at both time-points, global *Alms1* KO mice were severely hyperinsulinemic, with insulin concentrations around 10-fold higher than controls. Normoglycemic hyperinsulinemia was recapitulated in male *Alms1* MSC-KO mice at 19 weeks (Figure 2D).

Insulin and oral glucose tolerance tests (ITT and oGTT) were undertaken at 20 and 22 weeks of age respectively. These showed no reduction in hypoglycemic response to insulin in female *Alms1* global- or MSC-KO mice (Figure 2E,I) but increased glycaemic excursion after oral glucose in global KO mice, with a trend only in MSC-KO animals (Figure 2F,J). Both global- and MSC- KO female mice showed severe hyperinsulinemia (Figure 1M). Male global *Alms1* KO mice showed reduced insulin-induced hypoglycemia (Figure 2G,K), while MSC KO in males produced a trend towards reduction (Figure 2K). Global *Alms1* KO males showed unaltered glycaemic excursion after oral glucose (Figure 2H,L), but severe hyperinsulinemia (Figure 2N), and MSC-KO mice showed a trend towards increased glucose excursion and insulinemia on oGTT (Figure 2L,N). These findings demonstrate severe IR in global KO mice of both sexes, which is largely recapitulated in MSC KO mice. Hyperglycemia, however, was only prominent in female global KO mice, being only weakly echoed in female MSC KO mice.

The IR in AS is strikingly dyslipidemic [7], so we next determined lipid profiles. At 24 weeks, global *Alms1* KO mice were indeed strikingly hyperlipidemic (Table 1), albeit with a different pattern to human dyslipidemia, which features elevated serum triglyceride and suppressed HDL cholesterol. The global *Alms1* KO mice had higher serum total, HDL and LDL cholesterol in both sexes, but higher triglyceride in males only (Table 1). Male MSC-KO mice showed no lipid alterations, while female MSC-KO mice showed hypercholesterolaemia only. Free fatty acid (FFA) concentrations showed no genotype-related differences.

**Table 1:** Serum biochemical profiles of global and MSC-specific *Alms1* KO mice**.** Data are shown for high fat fed mice at 24 weeks of age. Global WT/KO and MSC WT/KO experiments were performed with identical design at different times. WT/KO comparison used an unpaired two-tailed Student's t-test with Bonferroni correction. For females N = 7, 8, 8 and 8 for global WT, global KO, MSC WT and MSC KO respectively. For males N = 8, 8, 7 and 7 for global WT, global KO, MSC WT and MSC KO respectively. p values below 0.005 (given 10 comparisons) are shown in bold.

	Males						Females					
	Global			MSC			Global			MSC		
	WT	KO	p value	WT	KO	p value	WT	KO	p value	WT	KO	p value
	Mean ± sd		WT vs KO	Mean ± sd		WT vs KO	Mean ± sd		WT vs KO	Mean ± sd		WT vs KO
FFA μmol/L	401 ± 69	464 ± 73	0.19	381 ± 78	598 ± 374	0.32	381 ± 35.5	507 ± 283	0.53	385 ± 76	383 ± 82	0.96
Cholesterol mmol/L	6.3 ± 0.8	8.4 ± 0.6	**<0.0001**	5 ± 0.7	6.1 ± 1.3	0.16	3.1 ± 0.5	7.2 ± 0.7	**<0.0001**	2.8 ± 0.7	5.2 ± 1.2	**0.0004**
Triglycerides mmol/L	0.8 ± 0.2	1.4 ± 0.5	0.011	0.7 ± 0.2	0.9 ± 0.2	0.11	0.8 ± 0.1	0.9 ± 0.3	0.25	0.8 ± 0.3	0.7 ± 0.2	0.94
HDL mmol/L	2.7 ± 0.3	3.3 ± 0.3	**0.0018**	2.3 ± 0.3	2.2 ± 0.8	0.88	1.5 ± 0.2	2.6 ± 0.3	**<0.0001**	1.2 ± 0.4	2.1 ± 0.4	**0.001**
LDL mmol/L	3.2 ± 0.6	4.5 ± 0.4	**0.0006**	2.4 ± 0.5	3.4 ± 0.6	0.012	1.3 ± 0.4	4.1 ± 0.5	**<0.0001**	1.3 ± 0.5	2.8 ± 0.9	**0.0024**
ALT U/L	105 ± 62	515 ± 215	**0.0002**	171 ± 209	636 ± 514	0.093	56 ± 15	494 ± 117	**<0.0001**	93 ± 112	225 ± 216	0.29
AST U/L	203 ± 188	468 ± 177	0.023	202 ± 179	696 ± 570	0.099	143 ± 64	553 ± 281	**0.0048**	269 ± 202	257 ± 174	0.90
Leptin ug/L	40.1 ± 11.6	41.2 ± 8.7	0.83	50.1 ± 15.5	74.4 ± 12.1	0.013	20.4 ± 12.7	63.1 ± 18.9	**0.0004**	18.9 ± 18.4	86.5 ± 10.5	**<0.0001**
Adiponectin mg/L	16.3 ± 1.3	8.5 ± 1.7	**<0.0001**	21.2 ± 1.7	15.2 ± 4.3	**0.0092**	28.8 ± 7	14.9 ± 2.3	**0.0002**	34.7 ± 8.2	28.7 ± 13.1	0.59
Testosterone ng/mL	2.0 ± 3.1	2.9 ± 4.9	0.66									

### 3.4 Effects of MSC-specific Alms1 deficiency on adipose cellularity are also sexually dimorphic

Consistent with the increased adiposity of female *Alms1* global- and MSC-KO mice, serum leptin concentration was increased in both. Male global *Alms1* KO mice appeared to show no difference in serum leptin concentration despite differences in fat mass, but this is likely artefactual, as leptin concentrations reached the upper limit of detection in the assay used. In keeping with this, a trend towards increased serum leptin concentration was seen in male *Alms1* MSC KO mice for which repeat assay of diluted samples was possible. Serum adiponectin concentrations were reduced in male and female global *Alms1* KO mice, and in male but not female MSC-KO mice. As adiponectin is suppressed by testosterone, and given male hypogonadism in AS, serum testosterone was assayed in male global *Alms1* KO mice, but was unaltered.

**Figure 3:**
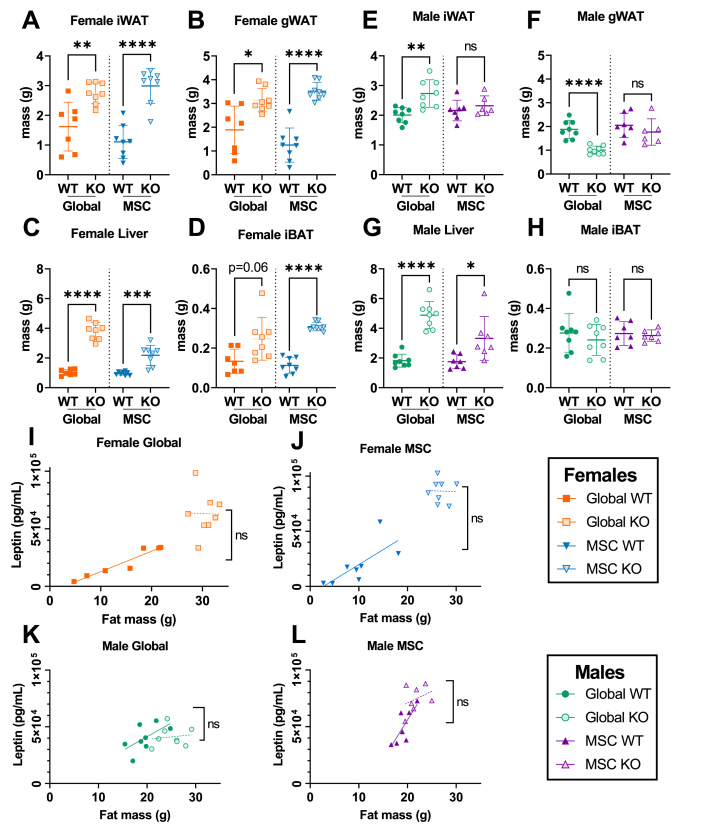
**Mesenchymal stem cell-specific *Alms1* knockout mice show hepatomegaly despite the absence of Cre-driven loss of Alms1 in hepatocytes, while fat pad mass is sexually dimorphic.** (A–H) Mass of inguinal, and gonadal white adipose tissue (iWAT and gWAT respectively), liver and interscapular brown adipose tissue (iBAT) of male and female mice at 24 weeks of age. (I–L) Linear regression of serum leptin concentration and lean mass of male and female mice at 24 weeks of age. Each data point represents an individual animal, with bars in (A–H) representing mean ± sd lines and lines in (I–L) representing lines of best fit. Comparison between WT and KO groups in (A–H) used an unpaired two-tailed Student's t-test with Bonferroni correction. Comparison between lines of best fit in (I–L) was performed by simple linear regression, with square brackets showing comparison of y intercepts. No significant change was seen between gradients. ∗ denotes p < 0.05, ∗∗ denotes p < 0.01, ∗∗∗ denotes p < 0.001 and ∗∗∗∗ denotes p < 0.0001. For females N = 7, 8, 8 and 8 for global WT, global KO, MSC WT and MSC KO respectively. For males N = 8, 8, 7 and 7 for global WT, global KO, MSC WT and MSC KO respectively. ns = not significant.

Having confirmed hyperlipidemic IR in global *Alms1* KO, as in human AS, and to a significant extent in MSC-KO mice, we then assessed mass and cellularity of adipose depots. At 24 weeks old, masses of inguinal and gonadal white adipose tissue (iWAT and gWAT) and liver were greater in female global and MSC-KO mice than controls (Figure 3A–D). Interscapular brown adipose tissue (iBAT) mass trended towards increase in global KO females, and was increased in MSC-KO female mice. WAT depot masses showed a divergent pattern in males. iWAT mass was increased but gWAT mass decreased in global KO, suggesting WAT redistribution. Neither change was recapitulated by MSC-KO (Figure 3E,F). No differences in iBAT mass were seen in males. When serum leptin concentrations were plotted against fat mass, no genotype-related difference was seen for either sex (Figure 3I–L).

**Figure 4:**
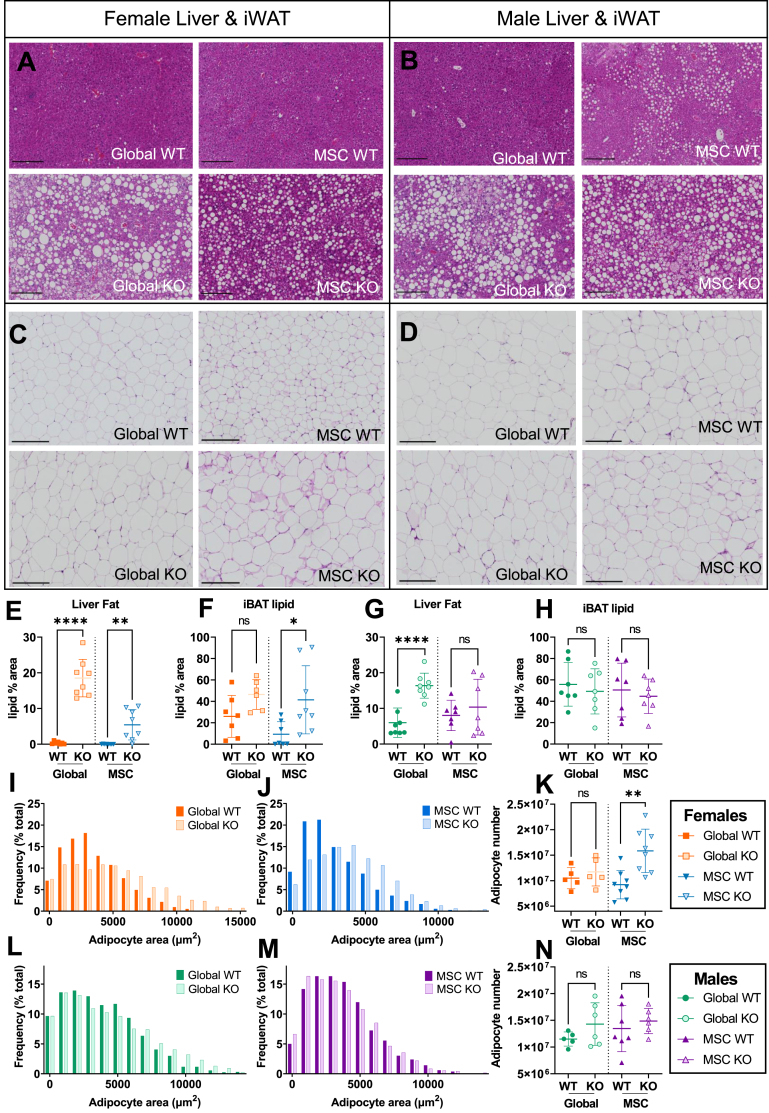
**Adipocyte hypertrophy and increased liver fat in both global and mesenchymal stem cell-specific *Alms1* knockout mice at 24 weeks of age.** Representative images of haematoxylin and eosin (H&E) stained liver and inguinal white adipose tissue (iWAT) sections from (A,C) female and (B,D) male mice. Scale bars 200 μm. Quantification of lipid content of liver (E,G) and interscapular brown adipose tissue (iBAT) (F,H) sections. (I,J,L,M) Size distribution of cross sectional area of adipocytes in iWAT, represented in bins of 1000 μm^2^ from comparisons of WT and global or conditional KO mice in separate experiments. (K,N) Total number of adipocytes in iWAT of each animal. Each data point in (E–H,K,N) represents an individual animal, with bars representing mean ± sd. Comparison between WT and KO in (E–H,K,N) used an unpaired two-tailed Student's t-test with Bonferroni correction. ∗ denotes p < 0.05, ∗∗ denotes p < 0.01 and ∗∗∗∗ denotes p < 0.0001. (E–H) For females N = 7, 8, 8 and 8 for global WT, global KO, MSC WT and MSC KO respectively. For males N = 8, 8, 7 and 7 for global WT, global KO, MSC WT and MSC KO respectively. (I–N) For females N = 5, 5, 8 and 8 for global WT, global KO, MSC WT and MSC KO respectively. For males N = 5, 6, 7 and 7 for global WT, global KO, MSC WT and MSC KO respectively. ns = not significant.

Cross-sectional adipocyte area was next measured for iWAT and gWAT (Figures 4C,D & S3A,B). Female iWAT and gWAT showed increased adipocyte size for *Alms1* global- and MSC- KO animals (Figures 4I,J & S3C,D) while iWAT of male global *Alms1* KO mice showed increased adipocyte size that was not recapitulated by MSC-KO (Figure 4L,M). Unlike all other WAT depots, male gWAT showed reduced adipocyte size in global *Alms1* KO (Figure S3F), but this was not seen on MSC-KO (Figure S3G). No change in calculated adipocyte numbers was seen for any depot except iWAT of female MSC-KO mice (Figure 4K,N & S3E,H).

### 3.5 MSC-specific Alms1 deficiency has lipotoxic effects on remote tissues

Adipocyte hypertrophy and dyslipidemic IR are consistent with an inadequate adipose hyperplastic response to positive energy balance. Such “adipose failure” characteristically leads to accumulation of lipids in remote organs, including liver and brown adipose tissue. Liver mass was increased in both global- and MSC- KO mice (Figure 3G), with pale macroscopic appearances of KO livers (Figure S4A and B) and macrovesicular steatosis (Figure 4A,B,E,G), although in male MSC-KO mice the difference in liver lipid did not reach significance. Serum ALT and AST concentrations, indices of hepatocellular damage, were increased in both sexes of *Alms1* global-, but not MSC-KO mice (Table 1).

iBAT lipid deposition, assessed by quantification of the proportion of the cross-sectional area accounted for by lipid droplets, tended to increase in female global KO, was significantly increased on MSC-KO (Figure 4F & S4C), but was unchanged in male global and MSC KO mice (Figure 4H & S4D). Collectively, these findings support the presence of ectopic lipid accumulation in global and MSC-KO animals, but again with some sexual dimorphism.

### 3.6 Alms1 knockout induces systematic meta-inflammation without adipose fibrosis or senescence

Widespread fibrosis of many organs, including liver and WAT, is reported in human AS, and is the target in an ongoing clinical trial [25]. It is unclear whether this mediates metabolic derangement and organ dysfunction, however, or is an epiphenomenon. Picrosirius red (PSR) staining of histological sections of liver, iWAT, and gWAT of global and MSC- *Alms1* KO mice revealed no significant changes in staining (Figure S5A–F), although a trend towards increase was seen in gWAT of female global KO mice.

Adipocellular senescence, both cell autonomous and paracrine, has been invoked as a contributor to obesity complications, and senolytic therapies are encouraging candidates to mitigate metabolic disease progression [26]. Given adipocyte hypertrophy, and expression of ALMS1 in the centrosome and mitotic spindle, we thus assessed senescence markers in iWAT and gWAT of global *Alms1* KO mice. However no difference in staining with Sudan Black B (SBB), which binds lipofuscin [27] (Figure S5G and H), nor immunohistochemical staining for p16, p21 and Lamin B1 (Figure S5I–N) was seen. We also determined serum concentrations of cytokines including constituents of the senescence-associated secretory phenotype (SASP) (Table 2). Female global KO mice showed marked elevation of core SASP components IL-6, and CXCL-1, and variable SASP components IL-10 and TNF-α, however IL-1β, one of the most reliable SASP markers, was unchanged. Male global KO mice showed a less striking profile of cytokines, although IL-6, CXCL1 and IL-10 were elevated. MSC KO mice broadly failed to reproduce the pattern of increases in cytokines seen in global KO animals, though female MSC KO mice did show increases in TNF-α and IL-6 of borderline significance. These findings support the presence of metinflammation in global Alms1 KO animals, and suggest that Alms1 expression in non *Pdgfrα* lineages such as immune cells may be involved in this. However, whether there is a significant burden of senescent cells in AS remains to be resolved definitively.

**Table 2:** Serum cytokine analysis in global and MSC-specific *Alms1* KO mice**.** Data are shown for high fat fed mice at 24 weeks of age. Global WT/KO and MSC WT/KO experiments were performed with identical design at different times. WT/KO comparison used an unpaired two-tailed Student's t-test with Bonferroni correction. For females N = 7, 8, 8 and 8 for global WT, global KO, MSC WT and MSC KO respectively. For males N = 8, 8, 7 and 7 for global WT, global KO, MSC WT and MSC KO respectively. p values below 0.006 (given 9 comparisons) are shown in bold.

	Cytokine	Males						Females					
		Global			MSC			Global			MSC		
		WT	KO	p value	WT	KO	p value	WT	KO	p value	WT	KO	p value
		Mean ± sd		WT vs KO	Mean ± sd		WT vs KO	Mean ± sd		WT vs KO	Mean ± sd		WT vs KO
Usually in SASP	IL-1β pg/mL	0.6 ± 0.3	1 ± 0.4	0.055	0.4 ± 0	0.7 ± 0.3	0.0306	0.5 ± 0.2	0.9 ± 0.6	0.27	0.5 ± 0.1	0.4 ± 0.1	0.75
	IL-6 pg/mL	9.7 ± 4.7	27 ± 8.5	**0.0004**	23.6 ± 14.4	36.2 ± 25.9	0.5674	9.9 ± 4.7	22.8 ± 4.5	**0.0002**	9.8 ± 4.6	25.8 ± 12.8	0.011
	CXCL1 pg/mL	131 ± 51	280 ± 120	0.0118	133 ± 57	283 ± 133	0.036	108 ± 37	306 ± 102	**0.0006**	117 ± 40	215 ± 142	0.16
Sometimes in SASP	TNF-a pg/mL	12.5 ± 4.5	26.2 ± 17.6	0.1012	12.5 ± 3.1	15.8 ± 6.5	0.4934	7.7 ± 1.5	19.4 ± 3.9	**<0.0001**	8.5 ± 1.1	13.9 ± 3.4	**0.0014**
	IL-10 pg/mL	22.4 ± 9.3	38.2 ± 12.1	0.0222	16 ± 4	25.9 ± 10.8	0.0882	28 ± 30.1	31.4 ± 4.1	**<0.0001**	16 ± 3.6	23.1 ± 7.9	0.074
Generally not in SASP	IFN-g pg/mL	0.7 ± 0.2	3.6 ± 5.7	0.34	0.6 ± 0.2	0.4 ± 0.1	0.0106	2 ± 2.6	0.8 ± 0.4	0.42	2.1 ± 3.9	0.5 ± 0.3	0.51
	IL-2 pg/mL	1.5 ± 0.5	2.2 ± 0.9	0.19	1 ± 0.3	1.2 ± 0.4	0.95	2 ± 0.8	2 ± 0.4	0.86	1 ± 0.2	2 ± 2.3	0.45
	IL-4 pg/mL	0.2 ± 0.1	0.4 ± 0.2	0.17	0.2 ± 0.2	0.2 ± 0.1	0.75	0.6 ± 0.4	0.5 ± 0.2	0.95	0.2 ± 0.1	1.4 ± 2.9	0.56
	IL-5 pg/mL	3.1 ± 1.9	4.3 ± 1.5	0.36	5.3 ± 2.7	5.5 ± 2.3	0.87	4.9 ± 0.7	7.2 ± 1.8	0.015	7.4 ± 4.1	10.1 ± 3.1	0.31

## 4 Discussion

The adipose expandability hypothesis holds that when the capacity of adipose tissue to expand by hyperplasia in response to positive energy balance is exceeded, adipocyte hypertrophy, necrosis and inflammation ensue, leading to dyslipidemic IR [28,29]. Adipocyte hypertrophy has been shown in patients with AS [13], and the dyslipidemic IR and fatty liver of AS strongly resembles the metabolic profile of lipodystrophies, extreme examples of “adipose failure”. We have thus suggested that loss of *Alms1* in the adipocyte lineage may be sufficient to explain many or all of the metabolic complications of AS [13]. We predicted that restricting *Alms1* KO to *Pdgfra-*expressing cells would abolish hyperphagia but induce severe IR, and fatty liver disease, despite intact hepatocyte *Alms1* expression. We also sought to determine whether *Pdgfra*-driven *Alms1* KO would modify the inflammation and fibrosis characteristic of liver and adipose pathology of AS [6].

The metabolic phenotype of the global *Alms1* KO mice we report accords with reported global KO models [[30], [31], [32]]. Some sexual dimorphism was seen, however. The greater metabolic derangement and obesity induced by *Alms1* KO in female compared to male mice, although not previously discussed, is consistent with the greater body weight increase reported in females than males in three prior *Alms1* KO mouse models [[30], [31], [32], [33]]. Some of this dimorphism may relate to different susceptibility of male and female WT C57BL/6 mice to diet-induced obesity. Females usually resist this more than males, potentially making pathologically increased susceptibility easier to discern [34,35]. No sexual dimorphism of metabolic traits has been reported in human AS, however the rarity of AS and lack of dedicated studies to date means that some sexual dimorphism of metabolic complications remains possible.

In keeping with our hypothesis that *Alms1* deficiency in the MSC/preadipocyte/adipocyte lineage accounts for the severe IR of AS, female *Alms1* MSC KO mice recapitulate the metabolic phenotype of global KO almost completely. Male *Alms1* MSC KO mice, although not exhibiting the obesity of *Alms1* global KO mice, also showed IR. The hepatic steatosis in female *Alms1* MSC KO is notable given intact hepatocyte *Alms1* expression, confirming that fatty liver in AS does not require hepatic *Alms1* deficiency. On the other hand, fatty liver is less severe in MSC-KO than global KO mice, and indeed in males there is no significant change. Moreover the increased serum ALT and AST of global KO mice is not seen on *Alms1* MSC-KO. This may implicate non *Pdgfrα*-Cre lineages in the hepatocellular damage that complicates fatty liver, but caution is warranted as global and MSC-KO animals were not studied in parallel. The greater fatty liver in female than male MSC-KO mice may also reflect the hyperphagia of female but not male mice, which would increase the load on adipose tissue.

Increased mass of iWAT and gWAT in female global and MSC-KO mice without increased adipocyte numbers is consistent with constrained recruitment of preadipocytes. Increased iBAT lipid is evidence of iBAT “whitening”, most likely due to lipid overspill from WAT. Neither adipose fibrosis nor histological evidence of adipocyte senescence were seen, though raised circulating cytokines indicate that metinflammation, and possibly senescence, may be at play systemically. This systemic inflammation was much less pronounced in MSC-KO mice, suggesting, as in liver, that *Alms1* loss in non *Pdgfrα* lineages may play a role in some sequelae of adipose failure. Decoupling of fibrosis from adipose failure and dyslipidemic IR provides evidence that fibrosis is not a prerequisite for these metabolic complications, and argues against anti-fibrotic strategies to treat metabolic complications of AS.

Male KO adipose tissue findings were more nuanced. Although a trend towards iWAT hypertrophy was seen, global *Alms1* KO males had decreased gWAT without hypertrophy, in agreement with the *Alms1*^*foz/foz*^ model [36]. iWAT showed no such reduction in our study or others [36]. Neither iWAT nor gWAT were changed in male *Alms1* MSC-KO mice, suggesting that the adipose redistribution in males is not accounted for by MSC-derived cells. Lineage tracing has shown that iWAT exhibits high levels of recombination with a *Pdgfrα*-Cre driver, while non-recombined adipocytes persist in gWAT [19]. It is not clear which sex these studies were performed in, but this raises the possibility of a methodological basis for our findings.

Generating *Adipoq*-Cre driven adipocyte-specific *Alms1* KO will be an important experiment to establish whether *Alms1* loss is causing adipose tissue failure due to effects on preadipocytes or mature adipocytes. This is a significant question given surprising prior data suggesting that restoration of *Alms1* function only in mature adipocytes of global *Alms1* KO mice ameliorates the adverse metabolic phenotype [13].

An unexpected finding in this study was that the hyperphagia of global *Alms1* KO was also seen in female – but not male – MSC-KO mice. Hyperphagia in *Alms1*^*foz/foz*^ mice precedes increased fat mass [31,37], while pair-feeding showed that hyperphagia contributes significantly to obesity and IR [37]. Such appetitive phenotypes are typically neuronally mediated, hypothalamic neuronal cilia have been implicated in appetite control [38], and *Alms1*^*foz/foz*^ mice have fewer hypothalamic ciliated cells [39]. We did not observe *Pdgfrα*-driven recombination in hypothalamic appetitive nuclei, however. Recombination was seen instead in the median eminence and elsewhere in a pattern consistent with oligodendrocyte precursor cells (OPCs), as expected given widespread use of *Pdgfrα*-Cre^ER^ to manipulate OPC gene expression. OPCs are thus strong candidate mediators of hyperphagia in response to *Alms1* KO. There is in fact growing evidence for a role for OPCs and their descendants in appetite control [23,40,41]. Mechanisms invoked include secondary loss of leptin receptors in the arcuate nucleus [40], and remodelling of perineuronal nets in response to nutritional fluxes in the median eminence [23]. Impaired differentiation of OPCs into oligodendrocytes due to impaired ciliary transmission of nutrition-related signals, leading to lower perineuronal net formation and less neuronal satiety signalling is a hypothesis worthy of examination in *Alms1* KO mice. OPCs are highly sensitive to sex hormones, and sexual dimorphism in OPC number, proliferation and migration is well established [42]. We speculate that this may explain the sexually dimorphic hyperphagia of MSC KO mice.

In conclusion, our findings indicate a key role for *Alms1* in adipocyte hyperplasia and thus buffering of positive energy balance. Loss of *Alms1* limited to MSC-derived cells including preadipocytes is sufficient to cause relative adipose failure on HFD, with secondary fatty liver and dyslipidemia despite intact hepatocyte *Alms1* expression. Surprisingly, our findings also raise the possibility that the hyperphagia of AS may be attributable to loss of *Alms1* in OPCs as well as, or instead of, neurones. Importantly, we found no fibrosis nor cellular senescence in adipose tissue or liver, despite severe systemic metabolic derangement. This argues against targeting these as therapeutic strategies in AS. Efforts to reverse positive energy balance are likely to be a higher yield approach to offloading failing adipose tissue and improving health.

## Funding

EJM is supported by a 10.13039/501100000274British Heart Foundation (BHF) PhD studentship [FS/18/57/34178], RKS by the 10.13039/100010269Wellcome Trust [210752] and the BHF Centre for Research Excellence Award III [RE/18/5/34216], and IL by the 10.13039/501100004359Swedish Research Council (2019-06422). This work was supported by the MRC MDU Mouse Biochemistry Laboratory [MC_UU_00014/5] and 10.13039/501100000268BBSRC [BB/V016849/1] to LKH. MGK and ZG are supported by grant 1R01DK125922 from the 10.13039/100000002NIH. For the purpose of open access, the author has applied a CC BY public copyright licence to any Author Accepted Manuscript version arising from this submission.

## CRediT authorship contribution statement

**Eleanor J. McKay:** Conceptualization, Data curation, Formal analysis, Investigation, Methodology, Project administration, Visualization, Writing – original draft, Writing – review & editing. **Ineke Luijten:** Supervision, Writing – review & editing. **Xiong Weng:** Investigation. **Pablo B. Martinez de Morentin:** Investigation, Methodology, Visualization. **Elvira De Frutos González:** Investigation. **Zhanguo Gao:** Resources. **Mikhail G. Kolonin:** Resources. **Lora K. Heisler:** Resources, Supervision, Writing – review & editing. **Robert K. Semple:** Conceptualization, Funding acquisition, Methodology, Resources, Supervision, Writing – original draft, Writing – review & editing.

## Declaration of competing interest

The authors declare the following financial interests/personal relationships which may be considered as potential competing interests:

Robert Semple reports a relationship with AstraZeneca Pharmaceuticals LP that includes: consulting or advisory. Robert Semple reports a relationship with Amryt Pharmaceuticals Inc that includes: consulting or advisory. Robert Semple reports a relationship with Eli Lilly and Company that includes: speaking and lecture fees. Robert Semple reports a relationship with Novo Nordisk Inc that includes: speaking and lecture fees. Robert Semple reports a relationship with Novartis Pharma AG that includes: consulting or advisory. Lora Heisler reports a relationship with AstraZeneca Pharmaceuticals LP that includes: consulting or advisory. Co-author LKH is an editor for Molecular Metabolism If there are other authors, they declare that they have no known competing financial interests or personal relationships that could have appeared to influence the work reported in this paper.

## Data Availability

Data will be made available on request.
