# Supplementary material for: Mesenchymal-specific Alms1 knockout in mice recapitulates metabolic features of Alström syndrome
Source: Mol Metab. 2024 Apr 6;84:101933. doi: 10.1016/j.molmet.2024.101933 (PMC11047791; doi:10.1016/j.molmet.2024.101933)
Supplement: Multimedia component 1 [file mmc1.pdf]

Supplementary Information for

Mesenchymal-specific Alms1 knockout in mice recapitulates key metabolic features of Alström syndrome

Eleanor J. McKay, Ineke Luijten, Xiong Weng, Pablo B. Martinez de Morentin, Elvira De Frutos González, Zhanguo Gao, Mikhail G. Kolonin, Lora K. Heisler, Robert K. Semple

Supplementary Table

Metabolite	Reagent manufacturer	Product code
Triglycerides	Siemens Healthcare	DF69A
AST	Siemens Healthcare	DF41A
ALT	Siemens Healthcare	DF143
Cholesterol	Siemens Healthcare	DF27
HDL	Randox	CH2849
FFA	Roche	Sigma 11383175001
Testosterone	Demeditec	DEV9911
Insulin & Leptin	MesoScale Discovery	K15124C-3
Adiponectin	MesoScale Discovery	K152BYC-2
Inflammatory cytokines	MesoScale Discovery	K15048D-2

Supplementary Table 1. Reagents used for serum biochemical asssays.

Supplementary Figures

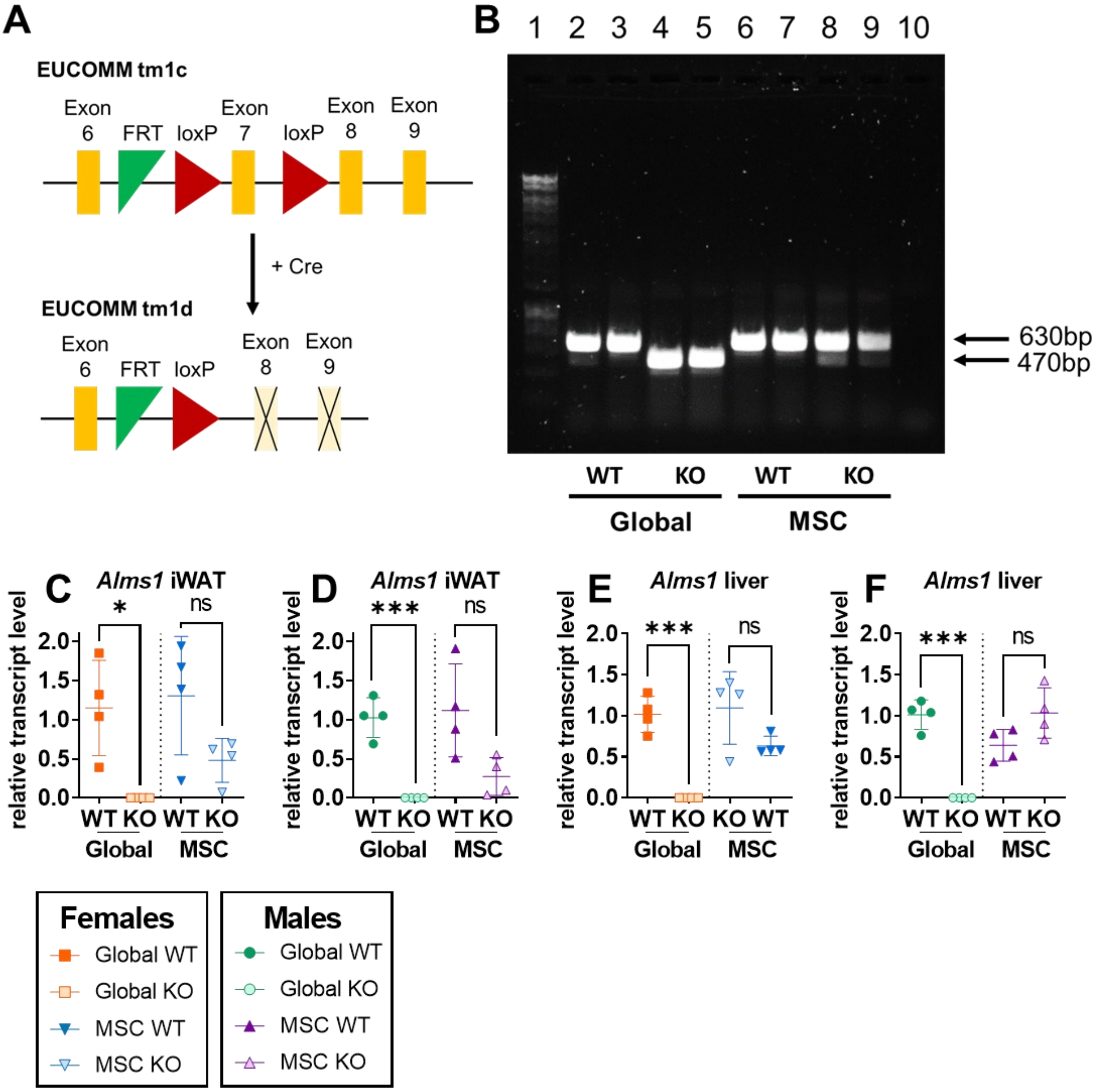

Figure S1. Validation of global and MSC-specific *Alms1* KO mice. (**A**) Schematic of *Alms1*^{tm1c} and *Alms1*^{tm1d} alleles, both generated from the EUCOMM *Alms1*^{tm1c} allele. (**B**) Liver cDNA gel electrophoresis following PCR amplification across area including exon 7 which is 160bp long. Lane 1: DNA ladder; lanes 2&3: *Alms1* global WT cDNA amplicon; lanes 4&5: *Alms1* global KO cDNA amplicon; lanes 6&7: *Alms1* MSC WT cDNA amplicon; lanes 8&9: *Alms1* MSC KO cDNA amplicon; lane 10, non-template control. (**C-F**) qPCR validation of *Alms1* loss in global and MSC-specific Alms1 KO mice using a Taqman probe spanning the exon 6-7 junction of *Alms1*, normalised to *Gapdh*. Global WT/KO and MSC WT/KO experiments were performed with identical design at different times, reflected in the dotted line separating the two cohorts. N = 4/group. Data represent individual animals with bars representing mean ± sd. Comparison between WT and KO in (**C-F**) used an unpaired two-tailed Student's t-test with Bonferroni correction. iWAT = inguinal white adipose tissue

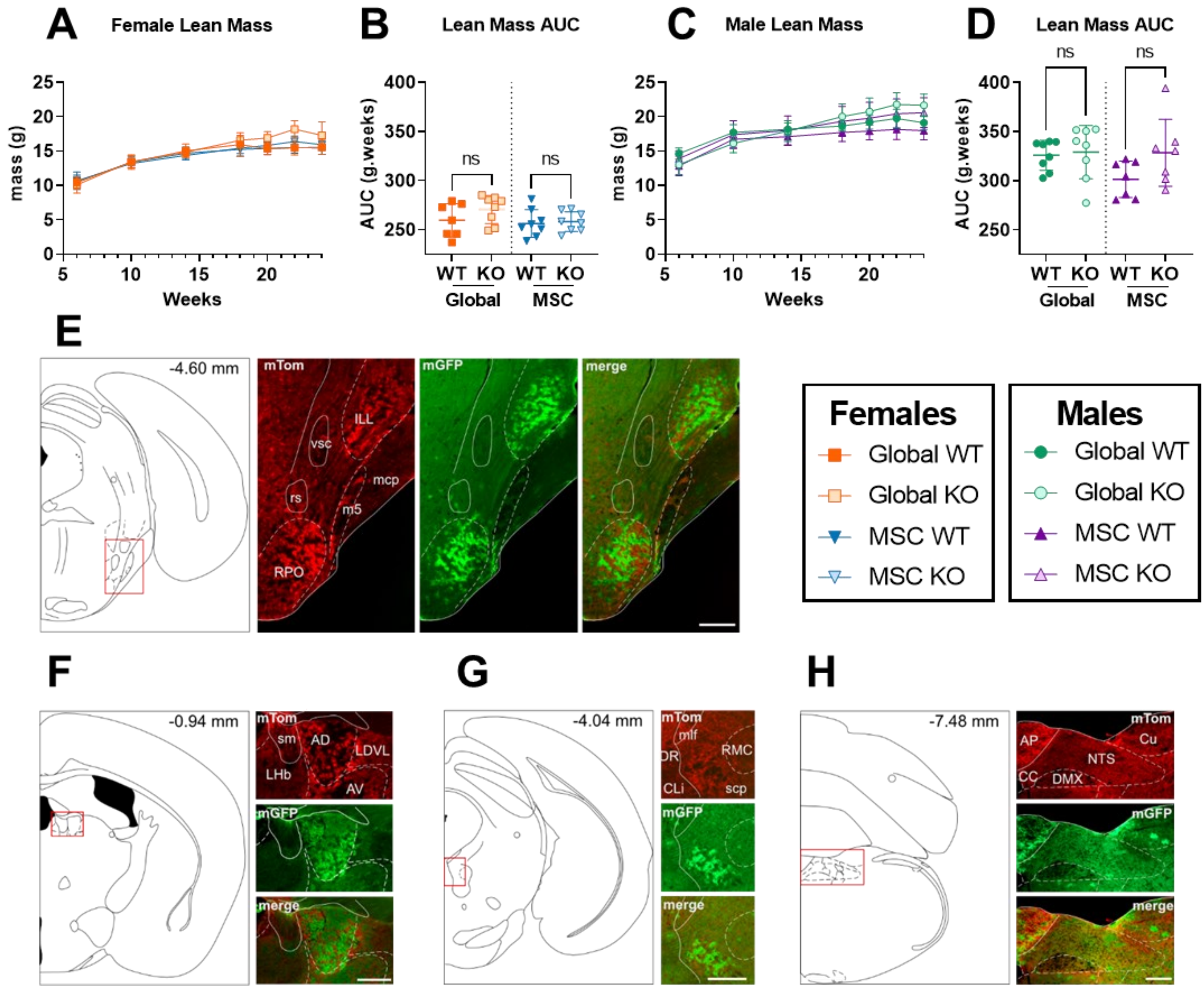

Figure S2. Female, but not male, MSC-specific *Alms1* knockout mice recapitulate the obesity and hyperphagia of global *Alms1* knockout. (**A-D**) Longitudinal analysis of lean mass assessed by td-NMR for female (**A**) and male (**C**) mice on high fat diet. For females N = 7, 8, 8 and 8 for global WT, global KO, MSC WT and MSC KO respectively. For males N = 8, 8, 7 and 7 for global WT, global KO, MSC WT and MSC KO respectively. AUC = area under curve WT/Global KO and WT/MSC KO experiments were performed with identical design at different times, reflected in the dotted line separating the comparisons. Longitudinal series plot mean ± sd for each time point. Data points in (**B,D**) represent individual animals with bars representing mean ± sd. Comparison between WT and KO in (**B,D**) was performed using an unpaired two-tailed Student's t-test with Bonferroni correction. **(E-H)** Illustration of coronal plane and representative microphotographs of brain sections from female Pdgfrα-Cre x mTom/mGFP mice showing expression of mTom, mGFP and merged images at bregma levels **(E)** -4.6; (**F**) -0.94; (**G**) -4.04; and (**H**) -7.48. AD: anterodorsal thalamic nucleus; sm: stria medullaris of the thalamus; AP: area postrema; AV: anteroventral thalamic nucleus; CC: central canal; CLi: caudal linear nucleus of the raphe; Cu: cuneate nucleus; DMX: dorsal nucleus of the vagus nerve; DR: dorsal raphe; LDVL: laterodorsal thalamic nucleus, ventrolateral part; LHb: lateral habenular nucleus; mlf: medial longitudinal fasciculus; NTS: nucleus of the solitary tract; RMC: red nucleus, magnocellular part; scp: superior cerebellar peduncle. Scale bar 200 µm.

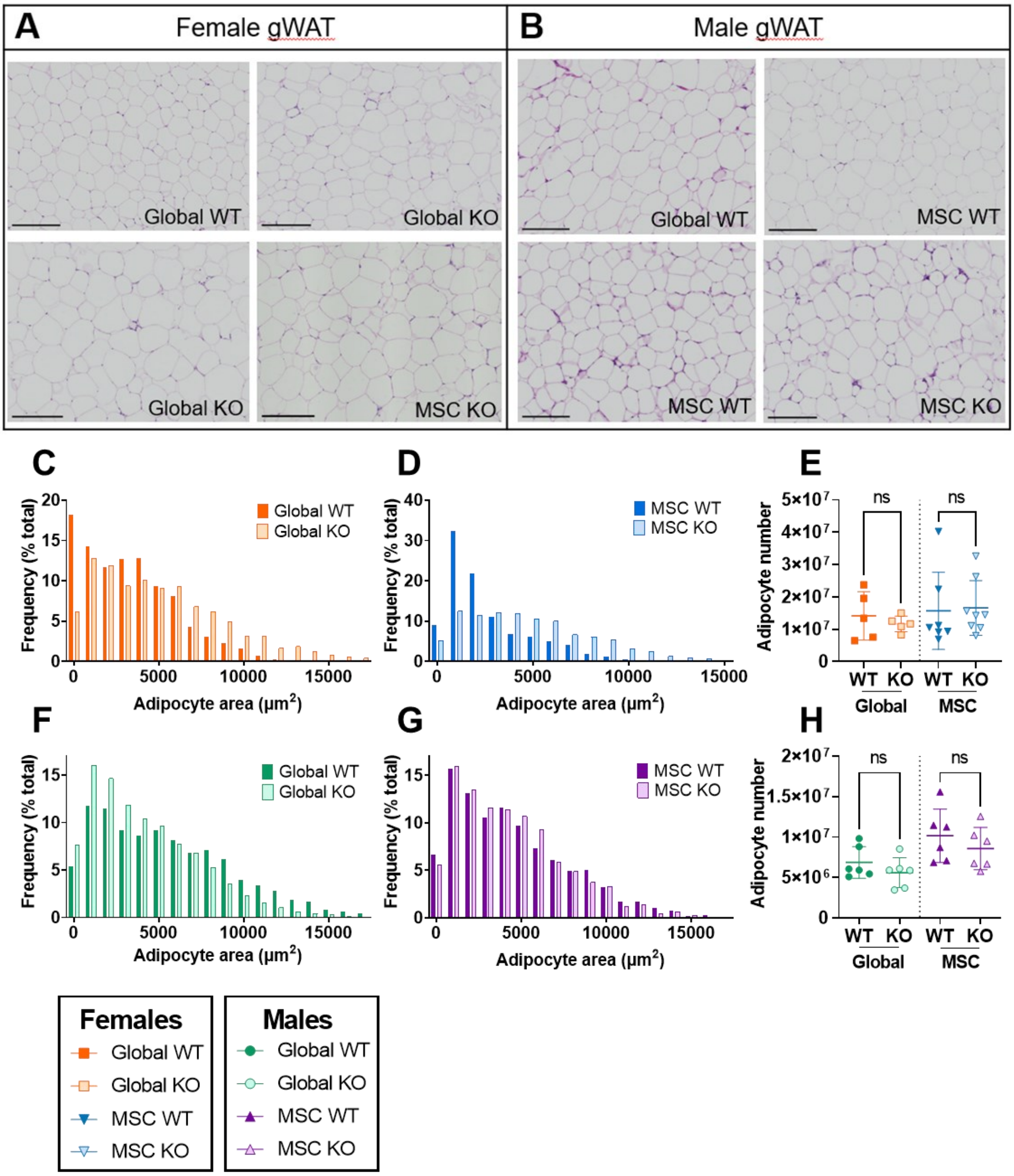

Figure S3. Histological evaluation of gonadal white adipose tissue of global and mesenchymal stem cell-specific *Alms1* knockout mice. (**A,B**) Representative microscopic images of haematoxylin and eosin-stained gonadal white adipose tissue (gWAT) sections from female and male WT, global or mesenchymal stem cell (MSC) knockout (KO) mice at 24 weeks of age on high fat diet. Scale bars 200μm. (**C,D,F,G**) Size distribution of cross sectional area of adipocytes in gWAT, represented in bins of 1000μm^2. (**E,H**) Calculation of the total number of adipocytes in the gWAT depot of each animal. Each data point represents one animal, with bars representing mean ± sd. Comparison between WT and KO groups in used an unpaired two-tailed Student's t-test with Bonferroni correction for multiple testing. (**C-H**) For females N = 5, 5, 8 and 8 for global WT, global KO, MSC WT and MSC KO respectively. For males N = 6, 6, 7 and 7 for global WT, global KO, MSC WT and MSC KO respectively.

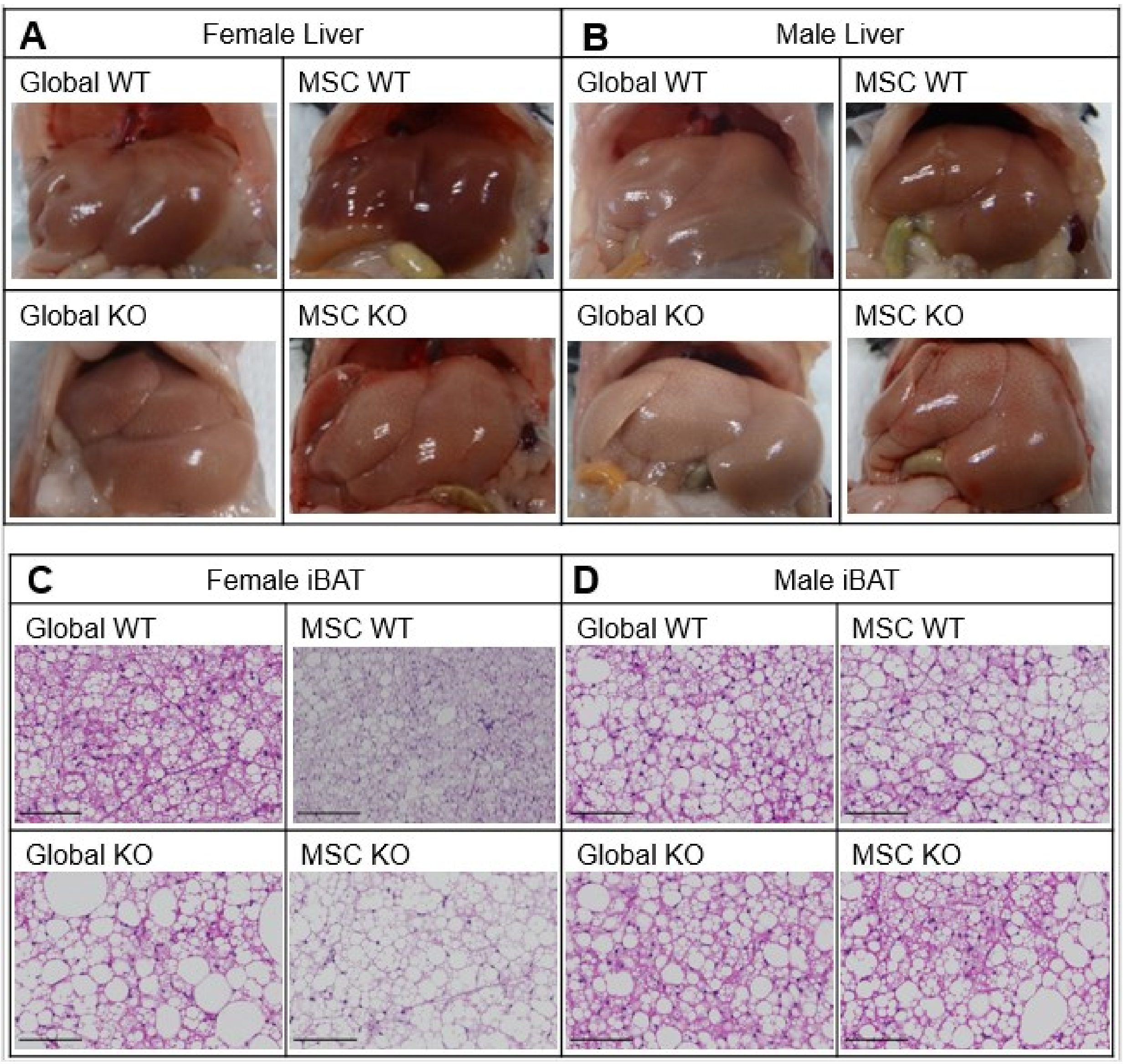

Figure S4. Histological evaluation of liver and interscapular brown adipose tissue of global and MSC-specific *Alms1* knockout mice. Representative macroscopic images of liver (**A,B**) and microscopic images of haematoxylin and eosin stained interscapular brown adipose tissue (iBAT) (**C,D**) from WT, global and mesenchymal stem cell (MSC) *Alms1* knockout (KO) mice at 24 weeks of age. Scale bars are 200µm.

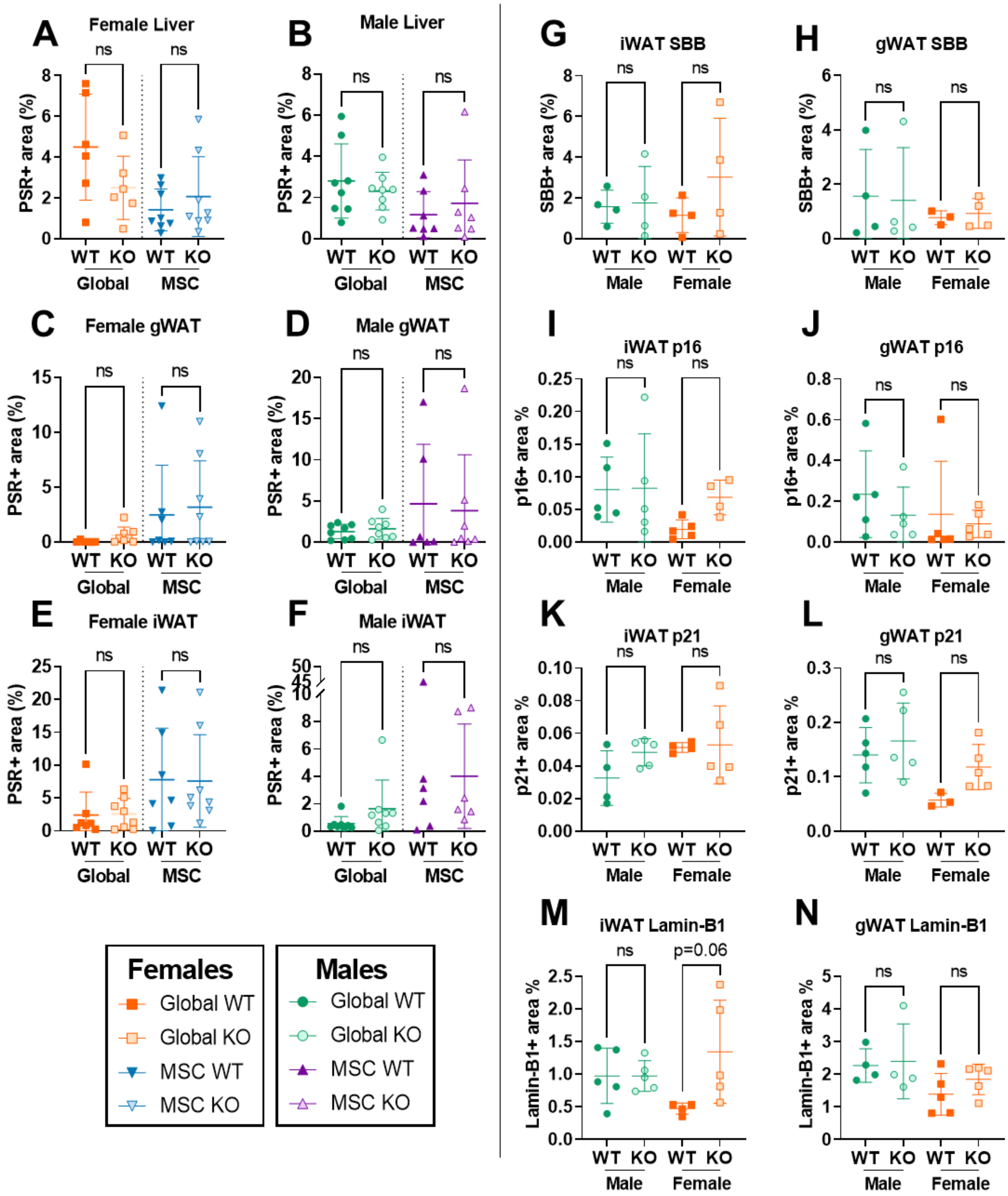

Figure S5. Histological evaluation of metabolic tissues from global and mesenchymal stem cell-specific *Alms1* knockout mice. Quantification of picrosirius red (PSR) fibrosis staining in liver (**A,B**), gonadal white adipose tissue (gWAT) (**C,D**) and inguinal WAT (iWAT) (**E,F**) in 24 week old male and female global and mesenchymal stem cell (MSC) knockout (KO) mice. (**G-N**) Quantification of senescence markers in gWAT and iWAT: (**G,H**) Sudan Black B staining for lipofuscin. (**I-N**) Immunohistochemical staining for (**I,J**) p16 (**K,L**) p21 and (**M,N**) LaminB1. Each data point represents one animal, with bars representing mean ± sd.

Comparison between groups in (**A-F**) used an unpaired two-tailed Student's t-test with Bonferroni correction. Comparisons in (**G-N**) used two way ANOVA with Tukey's multiple comparison test. * denotes p<0.05, ** denotes p<0.01 and **** denotes p<0.0001. (**A-F**) For females N = 7, 8, 8 and 8 for global WT, global KO, MSC WT and MSC KO respectively. For males N = 8, 8, 7 and 7 for global WT, global KO, MSC WT and MSC KO respectively. (**G,H**) N = 4/group. (**I-N**) N = 5/group.

Supplementary Methods

Animal studies

Alms1 Global- and MSC- KO mice were group-housed in individually ventilated cages (IVCs) at the Biological Research Facility at the University of Edinburgh where a 12-hour light/dark cycle (lights on 0700; off 1900) and controlled temperature/humidity (19-21°C/50%) were maintained. Prior to 6 weeks old, mice had *ad libitum* access to standard chow (CRM, Special Diet Service).

Glucose concentration was determined from the tail vein using the AccuChek Performa Nano [Roche, Switzerland]. Blood samples were collected in EDTA-coated capillary tubes and placed on ice before spinning at 4°C for 10 mins at 2000g and separating plasma. Plasma was stored at -80°C and thawed for biochemical analysis. Insulin was assayed by electrochemiluminescence immunoassay at the Medical Research Council Metabolic Diseases Unit (MRC-MDU) Mouse Biochemical Assay Laboratory at the University of Cambridge (Supplementary Methods).

Body length was measured using a digital calliper from nose-anus under anaesthesia at 24 weeks prior to cardiac puncture. At 24 weeks, a terminal bleed was undertaken by cardiac puncture under general anaesthesia and tissues were harvested immediately post mortem, weighed, and sectioned before fixing of tissues in 4% paraformaldehyde or snap freezing in liquid nitrogen.

Plasma biochemistry

Plasma biochemical assays, including cytokine assays, were undertaken by the Core Biochemical Assay Laboratory (CBAL) at the Cambridge University Hospitals NHS Foundation Trust. The Siemens Dimension EXL autoanalyser was used to quantify triglyceride, total cholesterol, HDL-C, alanine transaminase (ALT) and aspartate transaminase (AST). LDL-C is calculated from the triglyceride, HDL-cholesterol and total cholesterol concentrations as described in the Friedwald formula (LDL-C = Total cholesterol – HDL-C - (Triglycerides/2.2). Free fatty acids were quantified by enzymatic colorimetric assay and testosterone was quantified by enzyme-linked immunosorbent assay (ELISA); both reactions were measured on the PerkinElmer Victor-3 Plate Reader. Insulin, leptin, adiponectin and inflammatory cytokines were quantified by electrochemiluminescence immunoassay with measurements read on the MesoScale Discovery Sector s600. The reagent and kit details are detailed in **Supplementary Table 1**.

Histological studies

Paraformaldehyde-fixed tissues were paraffin-embedded and 5µm sections cut before hematoxylin and eosin (H&E) and picrosirius red (PSR) staining using standard procedures (1,2). Sudan Black B (SBB) staining was performed as described previously (3), but due to the fragility of adipose sections, wiping to remove excess stain was avoided. Immunohistochemical (IHC) staining for p21 (ab188224, 1:500, Abcam, UK), p16 (AHP1488, 1:500, BioRad, USA) and Lamin-B1 (ab16048, 1:100, Abcam, UK) was undertaken using a Leica BOND III automated immunostainer robot at room temperature together with staining with haematoxylin and 3,3′-Diaminobenzidine (H-DAB) using the Bond Polymer Refine Detection Kit. Liver and adipose histological slides were imaged using a Ziess Axioscan.Z1 with Zen2.6 software. Images at 20x magnification were analysed in QuPath v0.3.1 (4). SBB, and DAB staining in adipose and liver sections was quantified using an automated self-defined pixel classifier.

Adipocyte area was measured in QuPath after manual adipocyte identification using the wand detection tool. Typically this selected entire adipocytes, but some adjustments were required. At least 100 adipocytes were measured per adipose depot, a number sufficient for accurate representation of adipose depots (5). Adipocyte areas from QuPath were binned in GraphPad Prism into 1000μm^2 bins. Calculation of adipocyte numbers were based on assumed adipocyte sphericity, and an assumed adipose tissue density of 0.96g/ml (6), with depot masses divided by mean adipocyte mass to calculate total adipocyte number per depot. Lipid content and PSR, SBB, and DAB staining in adipose and liver sections was measured with an automated self-defined pixel classifier.

For the detection of mTom and mGFP in *Pdgfrα:Cre x mTom/mGFP* brains, the brains were dissected, post-fixed in formalin and cryoprotected with 30% sucrose at 4°C before coronal sectioning in 5 series at 25 µm using a freezing microtome (8000, Bright Instruments, UK). Sections were stored in anti-freeze solution at 4°C until processing. Dual immunohistochemistry was then performed using methods adapted from those previously described (7). Briefly, sections were washed with PBS with 0.2% Tween20 for 30 min and then PBS alone before blocking in 1% bovine serum albumin and 5% Donkey Serum for 1 hr at room temperature. Incubation with primary anti-RFP (rabbit, 1:1000, 600-401-379, Rockland Immunochemicals, USA) and anti-GFP (chicken, 1:1000, ab13970, Abcam, UK) antibody was for 24 hours at room temperature in blocking solution before washed with PBS with 0.2% Tween20 followed by PBS alone. Incubation with secondary antibodies (1:500, anti-rabbit AlexaFluor594, anti-chicken AlexaFluor488, Invitrogen, UK) was for 1 hr in blocking solution at room temperature. Of note, the usual permeabilization step with Triton X-100 was not used in any of the steps to avoid disruption of the cytoplasmic membrane. Images were acquired using Axioskope2 microscope and Axiovision software (Zeiss, Germany). All images were converted to 8-bit and merged using ImageJ (Fiji).

Gene Expression Analysis

Total RNA was extracted from 50 mg samples of liver and adipose depots by TRIzol™ according to the manufacturer's protocol. Concentration of eluted RNA was measured using the NanoDrop ONE before dilution to 100ng/µL in nuclease-free water. Reverse transcription was undertaken using the High Capacity cDNA Reverse Transcription Kit (Applied Biosystems) in the Eppendorf Mastercycler X50s, using 1000ng RNA per reaction. cDNA

solutions were diluted 1 in 4. Real-time quantitative PCR (RT-qPCR) was performed using TaqMan reagents including minor groove binder probes (8) on a LightCycler® 480 Instrument II (Roche). *Gapdh* was evaluated as a housekeeping control gene. Primer efficiency was calculated by standard dilution curve prior to experimental reactions, and reactions were run in triplicate with reverse transcriptase negative and non-template controls run on the same plate. Crossing point (Cp) values were calculated using LightCycler 480 software using the Abs quant/ 2nd derivative max function, and values for *Alms1* (probe Mm01189441_m1) normalised to *Gapdh* values after adjusting for primer efficiency, as described by Pfaffl (9).

Supplementary Methods References

1. Feldman AT, Wolfe D. Tissue processing and hematoxylin and eosin staining. *Methods in molecular biology (Clifton, NJ)*. 2014;1180:31-43. doi:10.1007/978-1-4939-1050-2_3
2. Bhutda S, Surve M V, Anil A, et al. Histochemical Staining of Collagen and Identification of Its Subtypes by Picrosirius Red Dye in Mouse Reproductive Tissues. *Bio-protocol*. 2017;7(21):e2592. doi:10.21769/BioProtoc.2592
3. Evangelou K, Gorgoulis VG. Sudan black B, the specific histochemical stain for lipofuscin: A novel method to detect senescent cells. *Methods in Molecular Biology*. 2017;1534:111-119. doi:10.1007/978-1-4939-6670-7_10
4. Bankhead P, Loughrey MB, Fernández JA, et al. QuPath: Open source software for digital pathology image analysis. *Scientific Reports*. 2017;7(1):1-7. doi:10.1038/s41598-017-17204-5
5. Parlee SD, Lentz SI, Mori H, MacDougald OA. Quantifying size and number of adipocytes in adipose tissue. In: *Methods in Enzymology*. Vol 537. Academic Press; 2014:93-122. doi:10.1016/B978-0-12-411619-1.00006-9
6. Entenman C, Goldwater WH, Ayres NS, Behnke AR. Analysis of adipose tissue in relation to body weight loss in man. *Journal of applied physiology*. 1958;13(1):129-134. doi:10.1152/jappl.1958.13.1.129
7. Wagner S, Brierley DI, Leeson-Payne A, et al. Obesity medication lorcaserin activates brainstem GLP-1 neurons to reduce food intake and augments GLP-1 receptor agonist induced appetite suppression. *Molecular Metabolism*. 2023;68:101665. doi:10.1016/j.molmet.2022.101665
8. Hein AE, Bodendorf U. Real-time PCR: duplexing without optimization. *Analytical biochemistry*. 2007;360(1):41-46. doi:10.1016/j.ab.2006.10.016
9. Pfaffl MW. A new mathematical model for relative quantification in real-time RT-PCR. *Nucleic acids research*. 2001;29(9):e45. doi:10.1093/nar/29.9.e45
